# The impact of the Wingate test on anaerobic power in the lower limbs of athletes with varied duration and load

**DOI:** 10.3389/fphys.2025.1582875

**Published:** 2025-05-14

**Authors:** Ying Liu, Juntao Yan, Zhigang Gong, Qi Liu

**Affiliations:** ^1^ College of Education, Beijing Sport University, Beijing, China; ^2^ Jiangxi Normal University Physical Education Institute, Jiangxi Normal University, Nanchang, Jiangxi, China; ^3^ General Administration of Sport Aquatic Training Monitoring and Intervention Key Laboratory, Jiangxi Normal University, Nanchang, Jiangxi, China

**Keywords:** anaerobic capacity, Winagte test, load, duration, surface electromyography

## Abstract

**Introduction:**

This study investigates the effects of Wingate anaerobic test (WAnT) on anaerobic capacity (AC) at different duration (20, 30, 40, 45 s) and resistance load (7.5%Body Mass (BM), 8.5%BM, 9.5%BM, 10.5%BM).

**Methods:**

12 male runners of 200, 400 m were subjected to four WAnT of different durations and loads. The changes of AC and lower limb muscle characteristics were analyzed by WAnT and electromyography.

**Results:**

The result shows that the integrated of electromyography (lEMG), root mean square% (RMS%), mean power frequency (MPF) and AC of lower limb muscles of rectus femoris (RF), biceps femoris (BF), vastus medialis (VM), vastus lateralis (VL), tibialis anterior (TA) and peroneus longus (PL) were increased by duration (P < 0.05 or P < 0.01), but there was no significant difference in lEMG of MG (P > 0.05). Load significantly increased AC, IEMG, RMS% and MPF of lower limb muscles (P < 0.01). The interaction effect of duration and load had no significant difference on PP, MP, IEMG, RMS% and MPF (P > 0.05), but increased on Fl (P < 0.05).

**Discussion:**

In conclusion, for 200 m and 400 m athletes, it is recommended to use a combination of 20 s and 10.5%BM load to achieve optimal peak power, as well as a combination of 20 s and 9.5%BM load for better average power. Different combinations of duration and load can be selected during the test to assess the corresponding capability.

## Introduction

Wingate anaerobic test (WAnT) is an effective method for evaluating anaerobic capacity (AC) with high repeatability (Castañeda-Babarro, 2021). The correlation coefficient of standard environmental conditions ranges from 0.89 to 0.98, and the test results are consistent with most AC results of the field, with a correlation coefficient of 0.75 or above, which has the advantages of objectivity and practicality (Zhou, 2009). At the same time, the WAnT test accounts for a large proportion of anaerobic energy supply, consistent with the power supply of sprint events, so it is also widely used in the international community to evaluate the anaerobic capacity of sprinters (Beneke et al., 2002; Yoshimoto et al., 2022; Cai, 2010). However, previous studies have shown that the optimal load varies depending on gender, project, and other factors. Therefore, the selection of different loads during testing will affect the accuracy of the evaluation results (Patton et al., 1985; Ozkaya et al., 2012; Jaafar et al., 2014; Pan et al., 2024). 30 s is the most widely recognized test duration for the Wingate test, yet some scholars still hold differing opinions regarding the test time (Calbet et al., 1997; Zhang and Guo, 2004). The optimal resistance load and exercise duration for the Wingate test to evaluate AC is not yet unknown.

In sprint events, previous studies on 100 m, 200 m, or 400 m races have compared AC at different durations under fixed resistance loads or compared AC at fixed durations under different resistance loads, the simultaneous study of duration and load variables is limited. For instance, Liu (2013) found that 200 m athletes showed significant AC in the 30 s and 400 m athletes in the 60 s anaerobic power test, while Chen et al. (2010) found that sprinters had relatively high AC in 10, 30, and 60 s WAnT. Zhang and Guo (2004) suggested that the optimal duration of the test should be selected based on the characteristics of the specific sprinting event, and recommended 40 s for the 400 m race. The typical load tested by WAnT is 8.5% body mass (BM), and several studies have shown that the appropriate load to produce the highest power output is between 10%BM and 11%BM (Jaafar et al., 2014; Gervasi et al., 2023; Liu, 2023; Üçok et al., 2005; Vandewalle et al., 1987a; Winter, 1991; Gordon, 1996). The likely reason is that a higher load recruits more fast-twitch muscle fibers, thereby generating greater peak power. Additionally, from biomechanical and metabolic perspectives, an insufficient load may fail to fully activate the anaerobic system, while an excessive load can reduce pedaling frequency, consequently diminishing power output (Antolinez et al., 2024). However, at present, there are few empirical studies on the optimal resistance load and exercise duration of 200 m and 400 m sprinters to test anaerobic capacity, and the test indexes are relatively simple. Sprinting requires high levels of ATP-CP and glycolysis energy supply, and athletes must move at maximum speed to cross the finish line as quickly as possible. The effect of load and duration on anaerobic capacity is mainly achieved by affecting the metabolic pathways and energy supply of muscles. During anaerobic exercise, the body mainly relies on the phosphocreatine system and the glycolysis system to provide energy. The increase in load will cause lactic acid accumulation in the muscles to accelerate and reach the lactic acid threshold, resulting in lactic acid accumulation in the muscles, which in turn causes fatigue (Spriet et al., 2000; Gaitanos et al., 1993). In addition, the larger load also causes the phosphocreatine reserves in the muscles to be depleted more quickly, limiting the duration of exercise. The increase in duration will make both the phosphocreatine system and the glycolysis system more fully utilized, especially the glycolysis system will become more important. Although the accumulation of lactic acid in the muscles will cause the glycolytic system to be more active and provide more energy, long-term intense exercise will also cause lactic acid to accumulate too much in the muscles, thus increasing fatigue (Mastalerz et al., 2024; Chatel et al., 2016). In conclusion, load and duration together affect the performance of anaerobic capacity.

It is indeed an urgent issue to determine the optimal exercise duration and resistance load that can accurately reflect the AC of 200 and 400 m athletes. Further experimental demonstration is necessary to address this challenge. The proposed study aims to explore anaerobic capacity under different duration (20, 30, 40, 45 s) and resistance loads (7.5%BM, 8.5%BM, 9.5%BM, 10.5%BM) in order to stimulate the maximum AC of the human body and enhance athletes’ athletic abilities. In addition, previous studies were mostly limited to the study of anaerobic power, and less involved in the discharge and fatigue of neuromuscles during exercise. Therefore, the analysis of athletes’ anaerobic metabolic ability and the changes in the electromyoelectricity generated by nerves during this process plays an important role in our understanding of the neuromuscular recruitment status during WAnT, and thus provides theoretical basis and feasible means for the evaluation of optimal anaerobic capacity.

## Methods

### Subjects

G*Power3.1 was used to calculate the sample size required for the experiment. With reference to previous studies, the effect size was set to 0.4, the test efficacy was set to 0.8, and the significance level was set to 0.05 (Teng et al., 2017). The sample size required for calculation was five people. This study randomly selected college athletes specializing in 200-m and 400-m events from local universities as the research subjects. They have over 5 years of training experience, constantly undergo high-intensity training, and have participated in several national competitions. The subjects were required to have no sports injuries within 1 month before the test and be in good health. During the intervention process, one person withdrew for physical reasons and two were absent from work. A total of 12 participants were ultimately enrolled, comprising 7 sprinters in the 200 m event and five in the 400 m (MacIntosh et al., 2003; Vargas et al., 2015). The experiment was conducted at the end of the preparation phase, when the athletes had developed a solid fitness base and were free from competitive commitments, allowing for better control of external variables (Boraczyński et al., 2020; Popadic Gacesa et al., 2009). The subjects are instructed not to consume alcohol or caffeinated beverages within 3 h before the test, and not to engage in high-intensity lower extremity training within 24 h before the experiment. Prior to the start of the study, all subjects were informed of the benefits and possible risks involved in participating in the study, and signed Subject Informed Consent after receiving a detailed explanation of the trial procedure. This study was approved by the Sports Science Experiment Ethics Committee of Beijing Sport University (2024104H) and was conducted in compliance with the principles outlined in the Declaration of Helsinki. The basic information of subjects is shown in Table 1.

**TABLE 1:** Basic Information of subjects (N = 12) (Mean ± SD).

Subject	Age (y)	Height (cm)	BMI (kg/m^2^)
Male college students	18.9 ± 1.0	177.7 ± 4.1	21.4 ± 1.6

### Measurements

In this study, a total of 16 Wingate tests were conducted using four different duration (20, 30, 40, and 45 s) and four different resistance loads (7.5%BM, 8.5%BM, 9.5%BM, and 10.5%BM). The final resistance value was calculated by multiplying the body weight of each subject by the resistance load. SPSS27.0 software was used to randomly group the test duration and load. Taking into account the recovery of the body after exhaustive exercise, the test was conducted twice a day in the morning and afternoon, at least 5 h apart, for a total of 8 days, and each test period was consistent with the previous day (Silveira-Rodrigues et al., 2021). All tests were conducted in an air-conditioned laboratory with a controlled temperature of 20°C and a relative humidity maintained at 60%–70% to eliminate the impact of environmental factors on the body. To analyze the effects of the Wingate test on anaerobic power and muscle characteristics of the lower extremities under different durations and loads, used surface electromyographic signal-related indicators. The time migration technology of Delsys wireless surface EMG system EMG server analysis software was applied to process the surface EMG and the raw EMG signals were bandpass filtered (10–480 Hz) and fully rectified (Liu and Huo, 2023). Subsequently, full wave rectification is carried out, and finally, the pre-processed EMG data is exported, analyzed and obtained.

### Procedure

#### Surface electromyography testing

According to the form of joint activity and muscle function, the anatomical analysis of lower extremity cycling and the search of relevant literature, the main muscle groups of hip, knee and ankle joint activity required for cycling were selected. Finally, seven muscle groups were selected: rectus femoris (RF), biceps femoris (BF), vastus medialis (VM), vastus lateralis (VL), tibialis anterior (TA), peroneus longus (PL) and Medial gastrocnemius (MG) (Li and Wang, 2015). To prepare for attaching the electrodes, it is recommended to disinfect the muscle anchor points with 75% alcohol first. Following this, remove any oil and cutin on the skin surface using a razor to minimize resistance. Subsequently, attach the electromyography electrodes parallel to the muscle fibers at the muscle belly.

#### Wingate testing

The experimenter informed the participants in advance of the testing methodology, essential movement techniques, and safety precautions during cycling. Before the test, the subjects were asked to do a standardized preparation consisting of 2 min dynamic stretching and 4 min fascia relaxation, followed by a warm-up on a power bicycle (Cyclus 2, Germany), during which the subjects cycled for 5 min and performed three quick sprints for 5 s, striving to achieve a heart rate of 130–140 beats per minute (bpm) (Mastalerz et al., 2024; Sauvé et al., 2024; Zupan et al., 2009; Li and Wang, 2015). The researcher, who also monitored surface electromyography (Delsys Tringo, USA), checked to see if the electromyography was normal. The formal experiment commenced with the experimenter giving the “go” command and asked to do their best to complete the test. The subjects were motivated by verbal encouragement in order to exercise as much effort as possible. The test follows a standardized protocol, starting from a static position with a preloaded resistance applied before the exercise test, followed by an all-out sprint within a specified time frame (MacIntosh et al., 2003).

#### Index selection

Peak Power (PP) refers to the capacity of muscles to generate highest power in a brief period, commonly known as explosive power. The peak power reflects the energy supply power of the phosphocreatine system, and the greater its value, the stronger the explosive force. Mean Power (MP) represents the average power output during 30 s of full motion, typically associated with speed endurance. The average power reflects the energy supply power of anaerobic energy supply metabolic system. The higher the average power, the better the speed endurance and the stronger the anaerobic capacity to do work. Fatigue Index (FI) assesses fatigue generation speed by measuring changes in power amplitude, reflecting the body’s anaerobic anti-fatigue ability (Jia and Wang, 2020). Integrated of electromyography (IEMG) refers to the total amount of motor unit discharge generated by muscle activities within a specific period of time. In other words, the size of integrated electromyography reflects both the number of motor units participating in muscle activities and the magnitude of each motor unit discharge, which is dependent on the amplitude value of electromyography. Root mean square (RMS) indicates the average muscle activation intensity over a certain period of time and is associated with the recruitment of motor units and synchronization of muscle fiber discharge. It serves as a representation of the magnitude of muscle force produced by muscles. In order to avoid excessive differences between individuals, the RMS value was standardized in advance. The standardized method adopted the maximum normalization method, which took the maximum RMS value of 16 Wingate tests on a single muscle of the subject as the standardized basic value, and the calculation formula was the RMS value of the test ÷ the maximum RMS value of a single muscle × 100% × 100% (Hernández-Belmonte et al., 2020). In subsequent context, RMS is replaced by RMS%. Mean power frequency (MPF) is the frequency over the center of gravity of the power spectrum curve. It can effectively explain the muscle activity and function state, and evaluate the degree of muscle fatigue (Shiming et al., 2016).

### Statistical analyses

The values obtained from the research test were expressed as mean ± sd. In this study, descriptive statistics will be used to analyze the basic information of the subjects, and two-factor repeated measurement analysis of variance will be used to compare the difference between anaerobic power index and myoelectric index of Wingate test at different durations and loads. If the results are significant, further pair-based comparison will be made. All pre-processed data obtained from the tests were statistically analyzed using Excel 2010 and SPSS 27.0 software. Before the two-factor repeated measurement ANOVA, the normal distribution was tested with this software, and all the data were consistent. The significance level was defined as P < 0.05, with a very significant level defined as P < 0.01, the effect size was calculated using partial eta squared (η^2^) to assess the magnitude of differences: <0.06 indicates a small effect, 0.07–0.14 a medium effect, and >0.14 a large effect. When calculating interaction effects, if the sphericity assumption is violated, the Greenhouse-Geisser correction is applied. GraphPad Prism 10.1 was used for mapping.

## Results

### Wingate test anaerobic power results at different durations and loads

**FIGURE 1:**
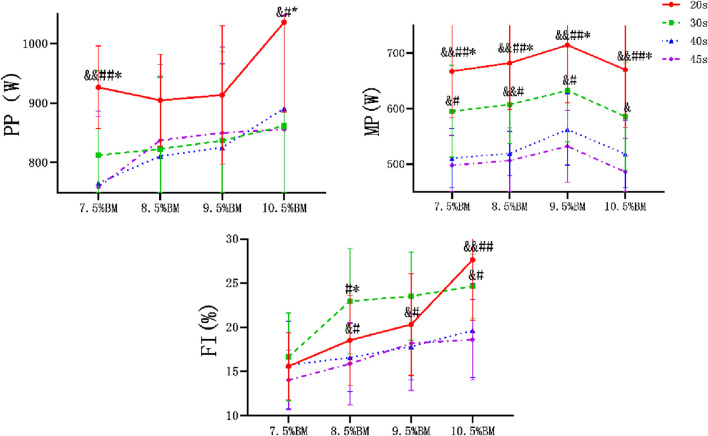
Wingate test comparison chart at different durations and loads. Note: Compared with 30 s: *p < 0.05, **p < 0.01; Compared with 40 s: ^#^p < 0.05, ^##^p < 0.01; Compared with 45 s: ^&^p < 0.05, ^&&^<0.01. The same below. PP, peak power, MP, mean power, FI, fatigue index.

After comparative analysis, it can be concluded from Figure 1 that the main effect of duration on PP was significant (P < 0.05, η^2^_PP_ = 0.163), and the main effect on MP, FI was extremely significant (P < 0.01, η^2^_MP_ = 0.524, η^2^_FI_ = 0.353). The main effect of load on anaerobic capacity was also extremely significant (P < 0.01, η^2^_PP_ = 0.353, η^2^_MP_ = 0.285, η^2^_FI_ = 0.629). The interaction effect of duration and load had no significant difference on PP [F_(5.67,83.15)_ = 1.759, p = 0.122, η^2^ = 0.107] and MP [F_(9,132)_ = 0.277, p = 0.980, η^2^ = 0.019], but had significant difference on FI [F_(9, 132)_ = 2.327, P < 0.05, η^2^ = 0.137].

### Electromyography results of Wingate tests at different durations and loads

**FIGURE 2:**
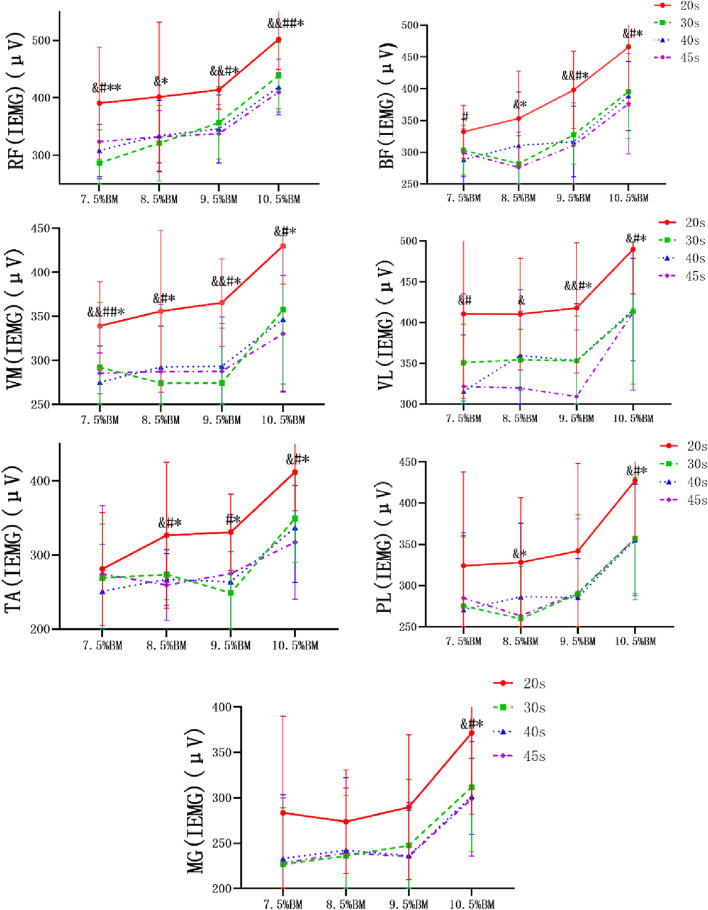
Wingate test IEMG comparison chart at different durations and loads. Note: RF, rectus femoris, BF, biceps femoris, VM, vastus medialis, VL, vastus lateralis, TA, tibialis anterior, PL, peroneus longus, MG, Medial gastrocnemius, The same below.

**FIGURE 3:**
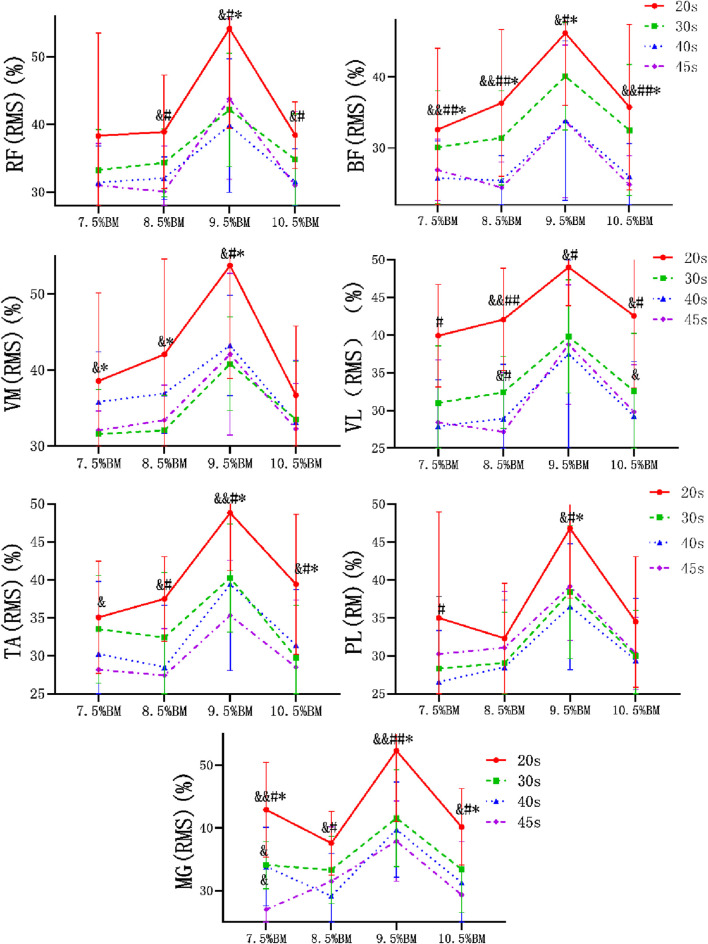
Wingate test RMS% comparison chart at different durations and loads.

**FIGURE 4:**
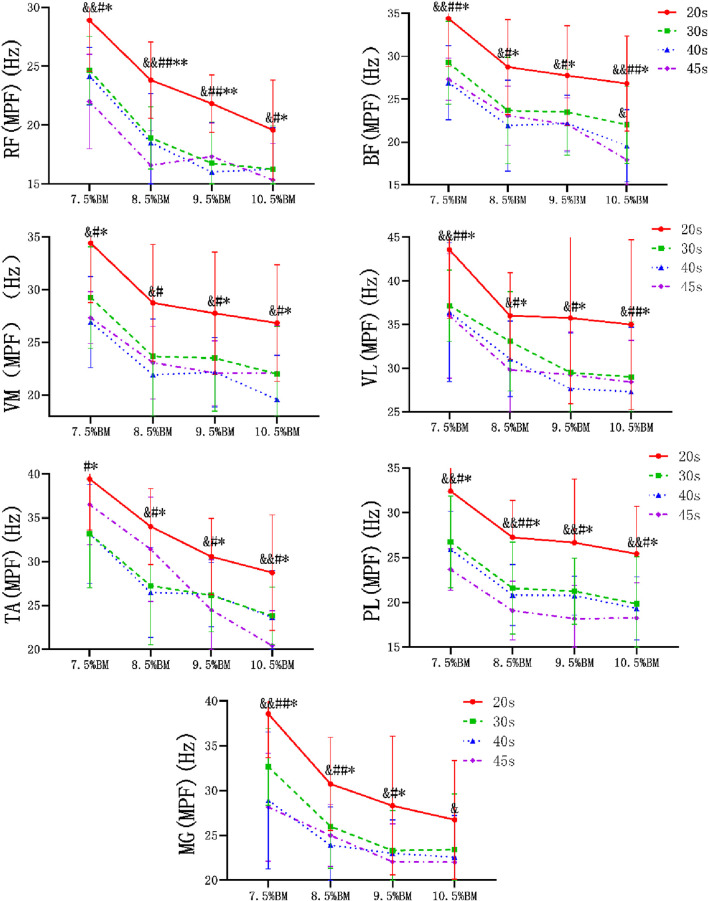
Wingate test MPF comparison chart at different durations and loads.

As can be seen from Figures 2–4, the main effect of duration on RF, BF, VM, VL, TA and PL was extremely significant, but the main effect on MG IEMG was not significant. The main effect of load on IEMG of lower limb muscle was extremely significant. The main effects of duration and load on RMS% and MPF of lower limb muscles were extremely significant. The interaction effect of duration and load had no significant effect on IEMG, RMS% and MPF of lower limb muscles. (Tables 2–4).

**TABLE 2:** Analysis results of the effects of time, load, and their interaction on IEMG.

Condition	Muscle name	df_1_	df_2_	F	P	η^2^
Duration	RF	3	44	14.105	<0.01	0.490
	BF	3	44	14.310	<0.01	0.494
	VM	3	44	14.823	<0.01	0.503
	VL	3	44	10.187	<0.01	0.410
	TA	3	44	7.138	<0.01	0.327
	PL	3	44	5.352	<0.01	0.267
	MG	3	44	6.895	>0.05	0.320
Load	RF	3	132	30.822	<0.01	0.412
	BF	3	42	20.345	<0.01	0.592
	VM	3	132	12.361	<0.01	0.219
	VL	3	132	12.691	<0.01	0.224
	TA	3	132	16.382	<0.01	0.271
	PL	3	132	13.058	<0.01	0.229
	MG	3	132	12.573	<0.01	0.222
Duration * Load	RF	9	132	0.522	0.857	0.034
	BF	9	132	0.738	0.673	0.048
	VM	9	132	0.415	0.925	0.028
	VL	9	132	0.343	0.959	0.023
	TA	9	132	0.853	0.569	0.055
	PL	9	132	0.153	0.998	0.010
	MG	9	132	0.151	0.998	0.010

**TABLE 3:** Analysis of the effects of time, load, and their interaction on RMS%.

Condition	Muscle name	df_1_	df_2_	F	P	η^2^
Duration	RF	3	44	7.645	<0.01	0.343
	BF	3	44	12.856	<0.01	0.467
	VM	3	44	5.621	<0.01	0.277
	VL	3	44	21.142	<0.01	0.590
	TA	3	44	8.112	<0.01	0.356
	PL	3	44	5.067	<0.01	0.257
	MG	3	44	17.234	<0.01	0.540
Load	RF	3	42	18.524	<0.01	0.570
	BF	3	132	16.868	<0.01	0.277
	VM	3	132	25.011	<0.01	0.362
	VL	3	132	17.600	<0.01	0.286
	TA	3	132	21.942	<0.01	0.333
	PL	3	44	21.270	<0.01	0.603
	MG	3	42	19.429	<0.01	0.581
Duration * Load	RF	9	132	0.620	0.778	0.034
	BF	9	132	0.399	0.934	0.026
	VM	9	132	0.949	0.486	0.061
	VL	9	132	0.192	0.995	0.013
	TA	9	132	0.804	0.613	0.052
	PL	9	132	0.375	0.945	0.025
	MG	9	132	1.459	0.170	0.090

**TABLE 4:** Effects of time, load, and their interaction on MPF.

Condition	Muscle name	df_1_	df_2_	F	P	η^2^
Duration	RF	3	44	28.199	<0.01	0.658
	BF	3	44	20.187	<0.01	0.579
	VM	3	44	22.477	<0.01	0.605
	VL	3	44	10.431	<0.01	0.416
	TA	3	44	13.100	<0.01	0.472
	PL	3	44	23.293	<0.01	0.614
	MG	3	44	13.217	<0.01	0.474
Load	RF	3	132	50.354	<0.01	0.534
	BF	3	132	24.763	<0.01	0.360
	VM	3	132	25.689	<0.01	0.369
	VL	3	132	15.982	<0.01	0.266
	TA	3	132	43.068	<0.01	0.495
	PL	3	132	20.344	<0.01	0.316
	MG	3	132	27.530	<0.01	0.384
Duration * Load	RF	9	132	0.745	0.667	0.048
	BF	9	132	0.335	0.963	0.002
	VM	9	132	0.221	0.991	0.015
	VL	9	132	0.206	0.993	0.014
	TA	9	132	1.505	0.152	0.093
	PL	9	132	0.077	1.000	0.005
	MG	9	132	0.730	0.681	0.047

## Discussion

The objective of this study was to explore the optimal load and duration combination for the Wingate test in 200-m and 400-m sprint events. Our results indicate that a 20-s duration demonstrated the best anaerobic capacity and muscle activation effects. In terms of resistance load, 9.5% of BM yielded better mean power output, while 10.5% BM exhibited superior peak power performance.

Some previous studies have investigated the relationship between 20 s WAnT and 30 s WAnT, and have concluded that there is a correlation between the WAnT values at these two duration intervals (Vandewalle et al., 1987b; Laurent et al., 2007; Vargas et al., 2015). Additionally, it was found that there was no significant difference in PP between the two duration intervals, but there was a significant difference in MP and FI. Moreover, it was suggested that there was no difference in the duration to reach PP and MP (MacIntosh et al., 2003; Astorino, 2011). However, it should be noted that the subjects in these studies were ordinary male college students or athletes from other sports, whose AC may differ significantly from that of 200 m and 400 m athletes, with the main training goal being the improvement of relative strength. In the present study, it was observed that both PP and MP showed higher levels at 20 s, but FI did not increase with the duration of the test. This may be attributed to the constant recruitment of muscle fibers during prolonged exercise to replace fatigued muscle fibers and maintain muscle strength for sustained pedaling. In high-intensity training, due to the high intensity of the training, muscles need to constantly generate greater strength, so type II muscle fibers are recruited first (Kraemer and Ratamess, 2004). This type of muscle fiber has a high glycogen storage capacity and strong creatine kinase activity, but low oxidase activity and is a typical anaerobic muscle fiber that is adapted to high-intensity anaerobic exercise (Gibala et al., 2006). Additionally, differences in anaerobic adaptation or muscle mass among subjects, as well as variations in fatigue-related metabolites, may have influenced performance (Vargas et al., 2015; Driss and Vandewalle, 2013).

Selecting the optimal load is crucial for accurately evaluating individual anaerobic power (Hargreaves and Spriet, 2020). The Wingate anaerobic power test has good external validity, as its average output power value has a high degree of negative correlation with sports performance in speed events. Üçok and Silveira-Rodrigues et al. conducted tests with different resistances and found no significant difference in MP between all loads (Üçok et al., 2005; Silveira-Rodrigues et al., 2021). However, the results of this study demonstrate that the average power of 9.5%BM is the highest at a given duration, which is inconsistent with previous findings. During exercise, the continuous supply of ATP is essential for muscle contraction in skeletal muscles, which is necessary for sustained movement (Hargreaves and Spriet, 2020). However, since the amount of ATP stored in muscles is limited, the metabolic pathway must be activated to maintain the required rate of ATP resynthesis during continuous exercise, which may decrease power output (Ekblom et al., 2018). On the other hand, the energy contribution from lactate produced anaerobically is insufficient to fully account for the PP variations, suggesting that hormones (epinephrine and norepinephrine) also play a significant regulatory role. Under high-intensity exercise, norepinephrine levels exhibit a positive correlation with exercise load (Gervasi et al., 2023; Ktenidis et al., 2021). This limited anaerobic reserve may also explain why the optimal load for MP is not the same as the optimal load for PP generation. 200 and 400 m athletes have more fast-twitch muscle fibers, greater strength, and are prone to fatigue. As force is reciprocal, the greater the load given, the greater the force that needs to be generated, and as a result, the fatigue index increases with the increase in load. This finding is consistent with the results of Silveira-Rodrigues et al. (Üçok et al., 2005; Silveira-Rodrigues et al., 2021). In addition, the study found that for the highest performing runners, higher maximum power and average power were generated during each test. Considering that the longer the duration of continuous sprint exercise, the higher the requirements for athletes’ anaerobic metabolic capacity and muscle fiber recruitment capacity. Therefore, compared with lower-level athletes, the higher-performing athletes have a higher glycolytic capacity, can produce more ATP through anaerobic metabolism, and have a stronger buffer capacity for blood and muscles. This is also consistent with the characteristics of their projects. In conclusion, this study found that the peak power of 20 s and 10.5%BM is the highest, and the average power of 20 s and 9.5%BM is the highest. The fatigue index reached its maximum at 10.5%BM.

Studies have demonstrated that the IEMG value of each muscle increases with the duration and load during continuous isometric contraction (Maton, 1981; Johnson et al., 1973; Jones et al., 2023). The study also found a significant enhancement in muscle discharge with prolonged duration and increased. Since the contraction speed of muscles will slow down with greater load, in order to maintain the work level of muscles, the lower limb muscles must mobilize more motor units to participate in the contraction, so the discharge energy of lower limb muscles will increase. This may be related to the phase change of the central nervous system’s stimulatory effect and the local muscle’s fast and slow muscle fiber recruitment response during exercise (Zhou and Yang, 1996). The stimulatory effect of the central nervous system is to compensate for the gradual decline in muscle strength, and the firing activity of motor neurons in the cerebral cortex is also gradually enhanced. This will inevitably further promote the excitatory effect on the spinal cord α motor neurons, so that the motor unit recruitment effect is strengthened, the muscle discharge is relatively increased, that is, in the continuous movement, due to the fatigue of some motor units, the body has to mobilize more motor units to participate in the work, or the discharge frequency of the same motor unit increases (Krustrup et al., 2004). Therefore, the iEMG value is increasing. In addition, studies have shown that the increase of IEMG is not completely linear with the change of load, but there is a surge period in a certain area, indicating that the sudden rise is caused by a large number of fast muscle recruitment, that is, equivalent to the strength of the anaerobic valve. This phenomenon is also evident in the present study. When the load increases to 10.5%BM, a significant increase occurs, which may be attributed to the rapid accumulation of lactic acid in local working muscles and the reduction of muscle blood flow caused by the load (Fatela et al., 2016). The study found that the athletes with better performance had greater discharge in the rectus femoris muscle, the medial femoris muscle and the lateral femoris muscle. The observed phenomenon can be attributed to the type II fiber predominance in these muscles, exhibiting biomechanical properties of fast-twitch contraction and high-force generation, albeit with compromised fatigue resistance (Kazuyukl, 2015). These muscles are also an important source of lower limb strength that affects the speed of elite sprinters.

Furthermore, the RMS value did not increase with the load, but reached its peak at 9.5%BM. The accumulation of metabolites can inhibit muscle activity. As the load increases, the level of muscle activation will no longer continue to rise to a certain extent, but will instead show a decreasing trend (Fatela et al., 2016). However, throughout the exercise process, as the duration of exercise extends, fatigue in major lower limb muscles deepens gradually. As muscle function status changes, each muscle’s activity level continuously adjusts and changes with increasing exercise duration. This may explain why the RMS value in this study differs from previous studies (Crenshaw et al., 1997). It can be seen from the results that the RMS value of the vastus medialis muscle is much higher than that of other muscles, indicating that in the whole sprint process, the vastus medialis muscle recruits more motor units to get greater muscle strength during the sprint process.

Some scholars have noted in their study on lower limb fatigue induced by exercise that MPF decreases as the duration of exercise increases, and the decrease becomes more pronounced with higher loads. However, the characteristics of muscle fatigue during lower limb muscle exercise exhibit different changes with varying forms of exercise (Xia, 2010). This is related to the ratio of fast muscle fibers to slow muscle fibers, fast muscle fibers conduct faster and discharge more frequently, while slow muscle fibers conduct slower and discharge less frequently. WAnT belongs to short-term high-intensity exercise, with the increase of exercise intensity, fast muscle fibers are first used for energy supply in this anaerobic exercise, so MPF will rise first, when fast muscle fibers fatigue, more muscle fibers including slow muscle fibers will be recruited for energy supply in order to maintain the exercise with high load, and then MPF value will decline. At this time, the myoelectric power spectrum moves from high frequency to low frequency. The 200 and 400 m athletes need a larger power output, and the fast muscle accounts for more, and the slow muscle is less. During the mobilization of fast muscle fiber, most of its energy comes from the anaerobic glucose metabolism, which will produce lactic acid, and a large amount of lactic acid will lead to muscle fatigue, so the MPF of each muscle basically showed a significant downward trend in the whole test (Piperi et al., 2024). The results of this study also indicate that as duration increases, noticeable fatigue occurs in all muscles and overall MPF exhibits a declining trend.

Nevertheless, several limitations should be noted. First, due to ethical and experimental constraints, this study only investigated anaerobic capacity and neuromuscular adaptations, without measuring blood markers or muscle fiber composition. Future research could further examine changes in blood lactate levels under different WAnT durations and loads, as well as their effects on the skeletal muscle system. Additionally, this experiment exclusively involved male athletes. Given the differences between male and female athletes in hormone secretion, energy substrate utilization, and fatigue resistance, the findings may not be generalizable to female athletes. Therefore, more studies are needed to evaluate anaerobic capacity in female athletes.

The development of anaerobic capacity is critical for success in numerous sports. As the most widely adopted anaerobic fitness assessment, WAnT yields findings from this study on male collegiate athletes that demonstrate enhanced event-specific relevance. These research outcomes can be effectively utilized by coaches and athletes to evaluate the anaerobic performance capabilities of 200 and 400 m sprinters.

## Conclusion

The Wingate Anaerobic Test, as the gold standard for assessing athletes’ anaerobic capacity, requires fine-tuned adjustments in test duration and load based on the sport-specific energy metabolism demands and neuromuscular adaptations. For events like the 200 and 400 m, which rely primarily on the phosphagen system for rapid energy supply while also depending on glycolytic system output for sustained performance, optimizing test parameters must account for both peak power and mean power. Research indicates that a 20-s duration effectively simulates the energy contribution timeframe of such events. A load of 10.5%BM enhances the force-velocity profile, allowing for more precise activation of type II muscle fiber recruitment, closely aligning with the physiological mechanisms of maximal power output in short sprints. Simultaneously, the higher mean power observed at 9.5% BM suggests that a slightly reduced load can prolong muscle fiber discharge duration, which corresponds to the metabolic demands for fatigue resistance during the latter stages of the event. This framework provides a practical approach for 200 and 400 m athletes, though long-term monitoring should be incorporated to optimize reliability and validity.

## Data Availability

The original contributions presented in the study are included in the article/Supplementary Material, further inquiries can be directed to the corresponding author.
